# ‘At Times it’s Too Difficult, it is Too Traumatic, it’s Too Much’: The Emotion Work of Domestic Abuse Helpline Staff During Covid-19

**DOI:** 10.1177/09500170231200080

**Published:** 2023-10-04

**Authors:** Chloe Maclean, Zara Brodie, Roxanne Hawkins, Jack Cameron McKinlay

**Affiliations:** University of the West of Scotland, UK; University of the West of Scotland, UK; University of the West of Scotland, UK; University of the West of Scotland, UK

**Keywords:** Covid-19, domestic abuse, emotional reflexivity, emotion work, helpline

## Abstract

During the Covid-19 lockdowns, domestic abuse helpline staff (DAHS) in the UK faced both a shift from working in an office to working-from-home and an increased demand for their services. This meant that during Covid-19, DAHS faced an increase in traumatic calls, and all within their own homes. This article explores the emotions work of DAHS to manage and work through their work-related emotions during Covid-19. Drawing on semi-structured interviews with 11 UK-based DAHS, this article suggests that working-from-home during the Covid-19 lockdowns amplified emotions of anxiety, helplessness and guilt for DAHS alongside an evaporating emotional distance between work and home life. Engaging in leisure activities and increased online meetings with colleagues were emotion work practices that DAHS used to emotionally cope. This article demonstrates that emotion work fills in for, and masks, the structural insufficiencies of employer worker-wellbeing practices.

## Introduction

The work of domestic abuse helpline staff (DAHS) is emotionally intensive. Domestic abuse helplines (DAHs) are mainly used by callers for response to a crisis, where DAHS listen to the traumatic and abusive experiences of callers, and signpost them to support services (Middleton et al., 2014). In March 2020, the UK government put in place the first ‘lockdown’ restrictions in response to the emergence of the Covid-19 health pandemic, which had implications for both DAHS and service users. The lockdown restrictions legally required the UK public to stay within their own homes unless they: were deemed an essential worker who had to work outside their home; had to purchase essential items such as food or medicine; required medical treatment; or were engaging in outdoor, individual or household exercise. During this period, the working environment of DAHS changed from shared office spaces to working-from-home (WFH) (Pfitzner et al., 2020; van Gelder et al., 2021).

Simultaneously, demand for DAHs increased during the lockdowns as many people found themselves ‘locked-in’ with an abusive partner or family member and without in-person domestic abuse services or in-person contact with external family or friends (Bradbury-Jones and Isham, 2020; Pfitzner et al., 2020). During the initial UK lockdown periods, calls to DAHs increased by up to 120%, and across the 10 months between April 2020 and February 2021, the average number of calls received by UK DAHs was 61% higher than the average of January to March 2020 (Refuge, 2021). The increase in DAH calls appears to mirror initial research, which suggests that the frequency of domestic abuse (DA) increased during the Covid-19 pandemic in 2020/2021 (Kourti et al., 2023; Petrowski et al., 2021). In this context, DAHS performed a critical emergency service during the Covid-19 pandemic (Bradbury-Jones and Isham, 2020; Petrowski et al., 2021).

Based on interviews with 11 DAH paid employees, this article explores the impact of Covid-19-related changes to DAH work, and how staff make sense of, manage and work through their emotions in relation to continual exposure to the traumatic experiences of callers. This article makes two key contributions to the existing literature. Firstly, it provides an insight into the changed work circumstances of DAHS in the UK during the Covid-19 pandemic. Secondly, it demonstrates the collective emotion work required to emotionally sustain DAHS. We argue that emotion work conducted within DAHs during Covid-19 fills in for, and masks, the structural insufficiencies of DAH worker-wellbeing practices.

## Literature review

### Emotions at work

Sociological explorations of emotions in the workplace often draw on Hochschild’s (2012[1983]) concept of emotional labour – workers’ management of their emotional expressions to meet the expectations, or formal regulations, of their workplace. This literature explores the ways in which management of staff’s emotional interactions with customers, co-workers or managers is used for commercial gains (Curley and Royle, 2013; Grandey et al., 2013; Harness et al., 2021; Tolich, 1993). In cases of not-for-profit organisations, such as social work and the National Health Service, the emotional labour of workers is conducted to maintain notions of professionalism that are underpinned by the contradictions of stoicism and care (Bolton, 2000; Kerasidou and Horne, 2016; Lavee and Stier, 2018; Lewis, 2005), rather than for commercial gain. Whether for commercial gain or the presentation of professionalism, emotional labour is a key concept used to explore the management of workers’ emotional performances.

Within the literature on emotions in the workplace, the term *emotional labour* is often used in a way that subsumes Hochschild’s concept of emotion work, obscuring the differences between the two forms of emotion management (Callahan and McCollum, 2002). Emotion work refers to how individuals attempt ‘to change in degree or quality an emotion or feeling’ (Hochschild, 1979: 551) where there is a discrepancy between emotions felt and emotions desired. This is a form of ‘deep acting’ that ‘goes beyond the mere ordering of display’ (Hochschild, 1979: 562) to induce a desired emotion. Emotion work is framed by ‘feeling rules’ – social rules that govern what are expected, desired, or accepted emotional responses within a given situation (Hochschild, 1979). Feeling rules enable us to understand emotional experiences as structurally informed by the social order.

Callahan and McCollum (2002) argue that the central difference between the concepts of emotional labour and emotion work is that the former provides an exchange value – emotional labour is done to create profit or earn a wage – while the latter is performed for its use value – for its functional use without an expectation to be compensated for it. While emotional labour is focused on interpersonal emotion performance in the workplace, emotion work is particularly useful for understanding the structurally informed intrapersonal emotion management of workers to cope with emotions experienced in the workplace.

We suggest that the concept of emotional reflexivity (Holmes, 2010) can be useful for understanding emotion work to cope with work-induced emotions. Emotional reflexivity enables us to explore the social and structural influence on emotional experience. It is the process of reflecting on our emotions to understand ourselves in relation to the social world around us (Holmes, 2010). The emotions involved in the reflective process include those that we feel, those expressed by others and those that we think we ‘should’ feel (Holmes, 2010) as set out by feeling rules. As such, we suggest that emotional reflexivity is a process that informs whether or how we engage in emotion work. This article will draw on the concepts of emotion work and emotional reflexivity to explore how DAHS in the UK managed their work-based emotions during Covid-19.

### Emotions in abuse work

Working with victims of abuse has been associated with emotional challenges. Such workers commonly experience emotions of anger, frustration, sadness, anxiety, guilt, hopelessness and loneliness (Crivatu et al., 2023). Most research exploring the emotional challenges of working to support victims of abuse has done so by exploring the emotional impact on staff in response to repeated exposure to stories of abuse and violence. For example, those working with victims or perpetrators of abuse have been found to experience secondary trauma (Benuto et al., 2021; Helpingstine et al., 2021; Morran, 2008; Ullman and Townsend, 2007). Secondary exposure to trauma has both physical and psychological impacts on those working with abuse survivors or perpetrators, such as sleep disturbance, headaches, intrusive thoughts related to the descriptions of abuse they have heard, depression and low morale (Benuto et al., 2021; Helpingstine et al., 2021). In a study of those working with victims of commercial sexual exploitation, secondary trauma was seen by staff as an inevitable part of their job (Helpingstine et al., 2021). Experiences of secondary trauma at work limit staff’s abilities to complete, or continue, their work (Ullman and Townsend, 2007) and are associated with higher rates of staff turnover (Figley, 1999).

Staff working with survivors or perpetrators of abuse have also been found to commonly experience ‘burnout’ (Babin et al., 2012; Crivatu et al., 2023; Helpingstine et al., 2021; Morran, 2008). Maslach (1982) suggests that burnout is a response to severe stress derived from ‘the chronic emotional strain of dealing extensively with other human beings, particularly when they are troubled or having problems’ (Maslach, 1982: 2). Burnout is suggested to have three core components: emotional exhaustion due to emotional demands; depersonalisation, whereby individuals objectify others to minimise the emotions that they feel in relation to others; and reduced personal accomplishment (Maslach and Jackson, 1981). In exploring the experiences of DA victim advocates, Babin et al. (2012) found that most advocates had experienced burnout at some point, and that burnout was more likely for those whose role involved frequent interactions with victims. While Morran (2008) also found burnout to be prominent among those working with male DA perpetrators, they found that men were more likely to feel energised to challenge and change perpetrators’ thoughts and behaviours, while women were more likely to feel emotionally exhausted and disillusioned (Morran, 2008).

Those working with survivors or perpetrators of abuse have also been found to experience feelings of anger and fear built through exposure to stories of abuse (Crivatu et al., 2023; Iliffe and Steed, 2000; Morran, 2008; Wasco and Campbell, 2002). Fear emerges when those working with survivors or perpetrators of abuse begin to see the victims as ‘like me’ and thus begin to see themselves as vulnerable to such abuse (Iliffe and Steed, 2000; Morran, 2008). The anger of those working with abuse survivors/perpetrators is often felt towards inefficient structures for preventing abuse (Iliffe and Steed, 2000), inefficient structures for supporting or bringing justice to victims (Wasco and Campbell, 2002) and perpetrators who demonstrate no remorse (Iliffe and Steed, 2000; Morran, 2008).

Abuse workers mitigate the negative impact on emotional wellbeing at work through a number of strategies. Abuse workers would often speak with their colleagues (Crivatu et al., 2023; Helpingstine et al., 2021) to remind themselves of positive people in their lives to contrast with the stories of abusers that they are exposed to (Morran, 2008), and engage in ‘self-care’ activities – doing something nice for themselves after an emotionally demanding shift (Helpingstine et al., 2021). Feelings of anger are understood by staff as a way of mitigating feelings of sadness from their work (Iliffe and Steed, 2000), increasing empathy and motivating them to address work-related challenges (Wasco and Campbell, 2002). At a managerial level, negative impacts on abuse worker wellbeing can be mitigated through high levels of organisational support, manageable workloads and opportunities for staff to discuss their emotions with peers or professionals (Babin et al., 2012; Crivatu et al., 2023; Helpingstine et al., 2021). The negative emotional impacts of working within the field of interpersonal abuse are also balanced by feelings of optimism for change (Morran, 2008), validation of the importance of their work (Crivatu et al., 2023; Helpingstine et al., 2021) and increased empathy (Wasco and Campbell, 2002).

### Critical work during Covid-19

Workers in critical support services, such as DA support, health and social care, experienced increased, emotionally intense workloads during Covid-19 (Cortis et al., 2021; Pfitzner et al., 2020; Roca et al., 2021; van Gelder et al., 2021; Vermeerbergen et al., 2021). Prior to Covid-19, health and social care was identified as emotionally intense work (Granter et al., 2019) that requires significant emotional labour to ensure patients feel cared for during distressing circumstances (Delgado et al., 2017; Kerasidou and Horn, 2016). Health staff experience burnout, in part due to the emotional intensity and demands for emotional labour in their work (Delgado et al., 2017; Granter et al., 2019). Health and social care workers faced increased service demand and increased suffering, including deaths, of those they supported during Covid-19 (Roca et al., 2021; Vermeerbergen et al., 2021; Weil, 2020). Such workers faced ‘moral injuries’ of being unable to help all who they would like to (Roca et al., 2021).

For DA workers specifically, the shift to WFH for indefinite periods during Covid-19 increased their workload as the demand for services increased and the modes of working changed (Cortis et al., 2021; Pfitzner et al., 2020; van Gelder et al., 2021). Many DA workers had to adapt quickly to providing online or telephone services during the lockdowns to replace in-person services (Cortis et al., 2021). Alongside this, many DA workers found it difficult to maintain boundaries between work and home, while WFH also led many to work longer hours (Pfitzner et al., 2020). Pfitzner et al. (2020) suggest that this meant the trauma of DA work entered workers’ homes, removing the feeling of home being a safe space away from trauma. The loss of communal working spaces also left DA workers feeling isolated and emotionally burdened (van Gelder et al., 2021).

While most research exploring DA workers’ emotions has focused on the emotional impact of their work, this article draws on Hochschild’s (1979) concept of emotion work to explore how DAHS comprehend and move on from the emotions such as sadness, fear, hopelessness and guilt evoked within their work during Covid-19.

## Methodology

To gain an insight into paid DAHSs’ experiences of their work during the pandemic, we conducted 11 semi-structured interviews with DAHS from across the UK. The interviews took place between February and May 2021. As such, the interviewees draw on experiences between March 2020 and April 2021, where areas in the UK experienced ‘stay-at-home’ measures for between six and eight months, and a variety of ongoing measures such as guidance to work from home where possible. Ethical approval to conduct the study was granted by the ethics committee of the University of the West of Scotland School of Education and Social Sciences.

### Participants

A diverse range of DAHs were approached that cater for a range of genders, sexualities, age groups and geographical locations within the UK. In total, eight DAHs were contacted and asked to send a research invite and participant information sheet to their staff. From this we recruited 11 participants from five different DAH organisations. Most participants were based in Scotland (*n* = 9), with one participant based in London and another participant identifying as being UK-based. Of the 11 participants, 10 self-identified as female and one identified as male, five DAHs were for women and children experiencing DA, three DAHs were for men experiencing DA and three were for any gender experiencing, or witnessing, DA. While this is a relatively small sample size, we determined that the sample provided sufficient ‘information power’ (Malterud et al., 2016). Information power refers to the ability of information gained through the sample to address the research aims, measured against the breadth of the research aim, sample specificity, quality of dialogue and whether cross-sectional or case study analysis is conducted (Malterud et al., 2016). Given the specific sampling criteria, and the quality of dialogue, the sample held the information power that enabled rich insights into and new knowledge of DAHS in the UK.

To protect the participants’ anonymity, they have been given pseudonyms. Participants and DAHs were made aware that DAHs would not be informed of whether a member of their organisation took part in the study or not, to further protect the anonymity of the research participants.

### Data collection

To minimise the risk of spreading Covid-19 and maximise the opportunity to interview participants near and far, interviews were conducted online via video conferencing software (MS Teams and Zoom). Online interviews have been found to increase participants’ comfort in discussing sensitive topics and can make the logistics of participating in an interview easier for participants (Jenner and Myers, 2019). Participants were asked to choose a day and time for the interview to ensure both a suitable time for the participant and to minimise the risk of family members being present during the interviews at home. The interview schedule explored four aspects of DAHSs’ experiences of their work during the Covid-19 pandemic: (1) how any changes in the quantity or content of calls impacted their work experience and wellbeing; (2) how WFH impacted their work experience and wellbeing; (3) what forms of support they received or were offered by their employer; and (4) what forms of support they received or sought outside of their employer to help manage work-related stresses. Participants were made aware that they did not have to answer any question that they did not want to and could stop the interview at any time should they feel upset. The interviewer paid attention to the emotional expressions of participants, and while many spoke passionately of their experiences, and some in a more detached manner, no participants appeared visibly upset or asked for a break in their interviews. Interviews lasted between 60 and 90 minutes.

### Data analysis

Thematic analysis was conducted by the research team, drawing on the six-step approach outlined by Braun and Clarke (2006). Each member of the research team was assigned two to three transcripts to familiarise themselves with and generate initial codes for interesting, re-occurring aspects of the data. Each researcher reviewed their initial codes to group-related codes in overarching themes. The research team then together reviewed the collection of initial themes and codes, considering the appropriateness of each: Is each theme unique? Do the themes resonate across multiple scripts? Is the theme relevant to addressing the research questions/aims? From this process, the research team agreed the following themes: worker wellbeing; disrupted work–life balance; and sources of wellbeing support. Each researcher returned to their assigned scripts to re-code based on the agreed themes and codes. Data from across the scripts were collated onto a thematic map.

## Findings and discussion

### Emotional exhaustion and moral injuries in DAH work

Participants described a number of emotional challenges when working on a DA helpline. In particular, the consistent exposure to traumatic experiences of callers was highlighted as evoking negative emotions, sometimes to the point of emotional exhaustion:

At times it’s too difficult, it is too traumatic, it’s too much. You know you can have five, six calls a day, that go on for longer than an hour, and then you expect somebody to just let all that go at the end of the day and come and do it again tomorrow. *Jack*

Some days it can really impact you, exhaust you or trouble you. *Kim*

The quote from Jack demonstrates his emotional reflexive process that draws out a tension between the expectations of his job – to listen to emotionally traumatic calls each working day – and his own emotional needs. Much like those who support victims of commercial sexual exploitation (Helpingstine et al., 2021), many participants highlighted that this form of emotional challenge was felt to be an inevitable part of their job prior to Covid-19. The feeling rules governing DAH work normalise exposure to trauma and require DAHS to be emotionally unaffected by this exposure.

DAHS pointed towards a sense of helplessness felt when listening to callers prior to, and during, Covid-19:

Sometimes we do feel quite helpless because women, for so many reasons, either will stay in the relationship or will go back, and people don’t understand about gaslighting and trauma . . .. *Susan*

The sense of helplessness felt by DAHS reflects ‘moral injuries’ (Roca et al., 2021) – injury to their sense of fulfilling the moral obligations of their work, or of their conscience. For DAHS, like healthcare workers (Roca et al., 2021), the consequences of being unable to help a caller may mean that the caller continues to experience physical and/or mental harm that can potentially be fatal. The degree to which DAHS felt helpless varied depending on how closely their helpline was connected to other services such as counselling, outreach services and refuges. The more closely DAHS were connected with other DA services, the less frequent or prominent discussions of feeling helpless seemed to be as DAHS felt able to direct callers towards help and support. However, the introduction of Covid-19 restrictions created disruption to such services, amplifying feelings of helplessness:

Not that I expect to solve everyone’s problems in one phone call, but you want to be able to offer something that’s useful . . . An emotional support – sometimes just having someone hear their truth and believe them is helpful. But there was something [in winter 2020] where there were a lot of calls that were . . . it was people who already knew what services and support was there, it was just a case of playing the waiting game until they got the services. I just found that quite difficult to kind of sit with. *Kim*

The reflections of DAHS above suggest that concern, helplessness and guilt were central emotions experienced in DAH work during Covid-19. While such emotions have been identified as part of the experience of working with survivors of abuse (Babin et al., 2012; Crivatu et al., 2023), these findings suggest that DAHSs’ awareness of the pandemic-related disruption to callers’ formal and informal support increased the moral injuries of DAHS, who were left to ‘sit with’ callers’ unresolved trauma. This echoes the findings of Pfitzner et al. (2020), who suggest that the initial Covid-19 lockdowns made DA support work feel ‘heavier’, with heightened concern for DA service users and increased exposure to the traumatic experiences of service users.

During Covid-19, the sense of emotional strain and exhaustion felt by DAHS was also magnified by their own personal emotional trials of understanding and living through the uncertainty of Covid-19:

In the first couple of months [of Covid-19 in the UK], you know, it was affecting my sleep. I was actually really anxious, and it was everything – it was the pandemic. But it was also work, too. *Lisa*

Emotional exhaustion inherent in the conditions of DAH work was exacerbated by DAHSs’ personal experience of Covid-19 ‘lockdowns’, the isolation that lockdowns involved and the fears generated by the pandemic. This intensification of negative emotions in an already emotionally dense form of work mirrors the experiences of other critical workers during the pandemic such as medical workers (Chaudhry et al., 2021) and social care workers (Vermeerbergen et al., 2021).The Covid-19 pandemic was a unique health threat with correspondingly unique public health policies – such as ‘lockdowns’ and ‘work-from-home’ regulations – that had not previously been experienced by those living in the western world. It created new everyday anxieties for DAHS that required new emotion work and emotion management strategies.

### Changed working conditions

In March 2020, all of the DAHS interviewed were required to work from home. At the time of interview, most interviewees had not returned to their workplace office. Those who had returned to their workplace office were allowed to do so on a one to two days per week basis only. Many DAHS experienced challenges in WFH that negatively impacted their own emotional wellbeing and emotion management strategies. The most frequently discussed challenge was the lack of a physical distance between work and home life:

You’re not able to, you know, leave that [work-based emotions] anywhere, and you’re on your own as well. So, you know, it can be very isolating . . . So, definitely, it is more difficult to deal with the challenging calls and to leave it at the work door because you were leaving it at the home door. *Sarah*

Work is in the same room that I have dinner and watch TV, you know. We might finish a call at 5 and then you’re just sitting yourself with that call [in your thoughts] for the rest of the day because there was no distraction. *Chloe*

The reflections of DAHS suggest that creating a physical distance between their work and their personal life was an emotion management strategy that Covid-19 WFH regulations disrupted, with a detrimental impact on their emotional wellbeing. The lack of physical distance between work and home life meant that work-induced emotions stayed with DAHS beyond the end of their working day, intruding into their personal life and ‘safe’ spaces. These findings resonate with the findings of Pfitzner et al. (2020) and van Gelder et al. (2021) who found that DA support workers struggled to switch off from work when WFH. They also support the suggestion of Drozdzewski and Dominey-Howes (2015) that there is a connection between emotions and places, whereby traumatic emotions become embedded within spaces. As such, for DAHS, WFH entails embedding traumatic work-based emotions within their homes.

The journey between home and work appeared to have previously provided a space for DAHS to perform emotional reflexivity to think through, and ‘let go’ of (Brownlie, 2014), worry, fear, or anxiety related to the calls that DAHS receive:

If you had a call that particularly stuck with you for whatever reason, you had that train journey to kind of process that and almost let it go out of your head . . . So usually by the time I got home it was out my head because in the meantime I’d be able to switch off or I’d met a friend on the train or whatever. And that’s obviously not there [at the moment]. *Kim*

As such, WFH restrictions made it more difficult for DAHS to perform emotional reflexivity to pause, or think through, work-related emotions at the end of their shifts.

WFH also reduced the space that DAHS had between their workplace and those they live with. Just as Pfitzner et al. (2020) found in their study of DA support workers in Australia, those who lived with family members had concerns about the possibility of their family members overhearing the traumatic nature of their DAH calls. DAHS identified strategies to minimise the likelihood of their family members, particularly children, overhearing their calls:

I’d call them [clients] back when the kids were in their bed at night. So, I would be doing phone calls at 8 o’clock at night. Really, just so the kids didn’t have to hear what we were talking about. *Rebecca*

In changing the hours she worked, Rebecca renegotiated the distance between her work and her family in an attempt to protect her children from overhearing the traumatic nature of her work. WFH in the same environment as their children created a ‘double burden’ for DAHS, whereby they had to manage and support the emotional needs of both their family members and DA survivors. Sometimes, these needs were in competition with one another:

I’d have a choice between answering that phone to a stranger and supporting that stranger, or supporting my daughter . . . That person on the end of the phone could be about to try and kill themselves, whereas my daughter is just upset because the computer is not working. So, it’s that kind of a fine balance. *Louise*

Such an experience has been common particularly for women shifting to WFH during the pandemic, where both work and family expectations collide (Rudrum et al., 2022). Owing to the critical nature of DAH work, the emotional tension in managing competing family and work expectations simultaneously is amplified.

The lack of physical separation between the workplace and home, alongside increases in service demand, additionally led to increased time spent working:

I find myself – selfishly in a way – looking at the clock when you’re in an office, and it’s quarter to 5, you’re going to think, ‘going to run on till half 5 if I make that call, but I’ve got other commitments’. If I’m sitting in the house and I know there’s nowhere else to go, then I’m more inclined to pick up the phone at quarter to 5 and not care if it runs on. So, it’s my own doing [working more]. *Una*

I found myself very quickly having to work much longer hours and unsociable hours just to manage the demand, and to think of strategies of how we can best address that. *Chloe*

Within the quotes above, feelings of guilt and duty to service users are central to the motivations for working overtime at home during Covid-19. Underpinning these feelings appears to be the feeling rule: DAHS *should* be happy to prioritise supporting clients beyond their working hours. Una’s quote demonstrates a conflict between the feeling rule and her expectation to work only her working hours, which leads her to interpret the expectation as ‘selfish’. It appears that the structural source of the feeling rule – within the culture of DAH work – is not recognised by Una, as she positions overworking as her ‘own fault’. The feeling rule thus encourages overworking, while masking adherence to the rule as an individual, rather than structural, problem.

The blurring of boundaries between home and work made it increasingly difficult to manage a sense of duty to callers and the personal life beyond work of DAHS. WFH during Covid-19 thus increased the workload of DAHS while limiting the physical and emotional distance between their work and home life.

### Managerial and peer emotion work

Colleagues and managers played an important role in assisting DAHS to think through, and move on from, work-induced negative emotions. Such support from colleagues and managers has been identified as critically important to those working with survivors of abuse (Babin et al., 2012; Crivatu et al., 2023; Helpingstine et al., 2021; Pfitzner et al., 2020) and in health settings (Lewis, 2005; Roca et al., 2021) both prior to and during Covid-19. WFH during Covid-19 entailed both additional resource needs, such as technical equipment to complete DAH work, and additional professional and emotional support needs, such as more frequent, formalised, debriefing sessions after difficult phone calls. DAHS who held managerial roles emphasised increasing the emotional support they provided their staff during Covid-19:

I think we’ve been very conscious about looking after each other a bit more, and particularly in a management role, I’ve been much more conscious of being a bit more ‘mother hen’ – ‘Is everyone alright? How’s things happening with whoever?’. . . We make things as flexible as we can. *Susan*

In the quote above, ‘support’ comes in the form of acknowledging the extra distress that their staff experienced during Covid-19, and opening up informal opportunities for staff to discuss their work-based difficulties. In doing so, managers can be seen to be assessing the emotional needs of their staff, and performing emotion work to attempt to mitigate negative emotions. In doing so, they also open up space for DAHS to engage in emotional reflexivity in conversation with them, whereby DAHS could think through the difficulties of their work. Many DAHS felt that their organisations’ management teams cared for, and supported, these needs:

My organisation has been really on the ball with making sure we have what we need, the equipment that we need, making sure that we are taking care of ourselves. I feel that my organisation personally, there’s not much more they can do. They’ve been really supportive, and they have let us know that if we need to take time off because of COVID then we can do that . . . they have kept our welfare at the centre of everything they do. *Kim*

She [the manager] was organising group meetings on Zoom, and we were having wee quizzes and stuff . . . We also got wee self-care gift bags through the post. So, like, wee chocolates and stuff. It was really nice. They have done that a few times. Things like that make you feel appreciated. *Rebecca*

DAHS appeared to view the support they received as: employers providing the resources and equipment to do their job that kept the service running, allowing staff to use their sick leave, creating online spaces to connect with their colleagues and providing ‘self-care’ gifts. Such support from management can be seen as multiple layers of emotion work practices that generated feelings of being supported and valued among DAHS WFH during Covid-19. Yet, most of this support was not new – such as sick leave, was necessary for the employer – such as providing equipment for WFH, or presenting the responsibility for emotional wellbeing as an individual responsibility – such as self-care gifts. The support highlighted by DAHS did not address issues of overworking or trauma entering their homes.

Not all DAHS felt supported by their organisations during Covid-19. Some DAHS highlighted a lack of formal support for dealing with DAH calls that may have exacerbated negative emotions:

Well, there was no support. And that was one of the reasons that prompted me to leave, there was a lot of responsibility and a lot of stress and a lot of traumatic things being disclosed to you and never unpacking them with someone. *Una*

This lack of emotion care led to increased feelings of uncertainty, helplessness and stress. For some participants, this lack of support existed prior to the pandemic too. However, Covid-19 WFH conditions, alongside increased demand for the DAH services, made it even more difficult to access organisational support. For those who had a less supportive management team, peer support in the form of formal debriefs or informal conversations were deemed particularly important. WFH during Covid-19 made informal peer-to-peer support, in particular, more difficult, or absent:

Just having colleagues around when you’re in the office, if you have a bad call they can hear how you’re talking so they can hear that you’re having that bad call, so they can talk nonsense to you after the call if that’s what you want, or they can talk through it with you. Whereas when you’re in a room on your own (at home), they’re not hearing that. *Louise*

The reduction of conversations with peers stalls the emotional reflexive process to think through the emotions evoked from difficult calls, and reduces opportunities for colleagues to perform emotion work to reframe the emotional experience. The loss of such conversations was highlighted as particularly detrimental to their work experience, and ability to cope. This finding is echoed by Pfitzner et al. (2020) and van Gelder et al. (2021) in their research on DA support workers’ experiences during Covid-19 in Australia and The Netherlands, respectively.

WFH revealed how important collective emotion work interaction is to support dynamics. In this section, we have highlighted the web of emotion work participated in, or not, by managers and colleagues to mitigate the negative emotions of DAHS, and to enable them to continue their work. This suggests that workplace emotion management is a collective dynamic, which the isolation of WFH threatened.

### Emotional coping strategies

DAHS developed a number of strategies to cope with the emotions experienced through their work during the Covid-19 pandemic. These strategies are a form of emotion work (Hochschild, 1979) – of attempting to replace, or minimise, an undesired emotion. While many DAHS mentioned meeting friends as a key coping mechanism for dealing with the pressures of their work, at times the Covid-19 restrictions limited their ability to do so. In turn, this interrupted the pre-lockdown emotional coping mechanisms of DAHS:

Depending on where you’re living and what the measures were, you couldn’t just go to your friend’s house. And, although we don’t talk about our work or the nature of our work because it’s confidential, you know, you would be able to just go around and say to someone, ‘Oh, I’ve had a bad day, let’s just have a chat’. And you can kind of do that over the phone, but it’s not the same. *Kim*

Many DAHS increasingly used leisure activities to ‘unwind’ or shift their focus away from the stresses of their work during periods of lockdown restrictions:

I found, especially when things are really hard (due to the increase in workload during the pandemic), it was very important to get out for that daily walk. And I was trying to do that, like, just be in nature. I live near hills, so I could do that . . . I was doing daily, like, yoga classes when things were really hard. *Lisa*

I found that going on the walks and, you know, having that decompression time, didn’t really help. It just made me overthink . . . Whereas, actually, if you just put the phone down at the end of the day and you go, you know, play Grand Theft Auto or whatever, that experience is the distraction. That experience gets rid of it [emotions or concerns about work]. *Jack*

I have been sitting in silence – I guess you could call that mindfulness, or you know some kind of meditation – but just having some complete silence to switch off from work. *Chloe*

Meditation-based practices, gaming and physical activities were all highlighted as mechanisms for overriding, or pausing, emotional reflection of their work-related issues, by providing a distraction from them (Brownlie, 2014). Such leisure activities demand attention, and create new emotional experiences that can pause, or replace, the negative emotional experiences (Maclean, 2021) of work. Here we can see emotion work is the work to pause or stop emotional reflection. Most participants who used these coping mechanisms did so prior to Covid-19. However, Covid-19 challenged DAHS to creatively find new, or adapt, coping mechanisms due to restricted use of other coping mechanisms (such as visiting friends), and/or due to the intensified workload of DAHS.

DAHS would also engage in comparative reflection as a way of coping with their work both pre and during the pandemic. In this process, DAHS would come to accept their own struggles at work by thinking through their experience in relation to that of their clients or others deemed to be in unfortunate positions:

One thing that [work] is good for is grounding. It can actually make you finish a shift and just go ‘actually, my life is okay because I’ve got a partner and a cat, it’s pretty boring’ (*laughs*), and I quite like it that way. So, not that you should use other people’s misfortune to feel better about yourself . . . but it kind of . . . actually, I’m okay, my life is okay. *Kim*

I think there’s so much to be said for keeping our perspective and keeping, sort of, you know, gratitude for what you do have, because I’ve said to my team quite a few times, ‘Let’s just remember that we’ve all kept our jobs’. *Susan*

In the quotes above, DAHS appear to feel compelled to comply with a feeling rule ‘I ought not to feel bad about my life because others’ lives are worse’. In assessing their own emotional wellbeing in comparison with their callers or those who have lost their jobs during the pandemic, DAHS have both engaged in emotional reflexivity and performed emotion work, whereby reflections are used to attempt to create positive feelings of gratitude to replace negative emotions felt within their work. Reflexively creating a comparative distance to their callers provided a counter to feelings of fear and helplessness that develop through identifying similarity with victims of abuse (Wasco and Campbell, 2002). In comparing themselves to their callers, the bar of acceptable emotional wellbeing was set low (Pfitzner et al., 2020), but, none-the-less, worked to enable some DAHS to move on from feelings of anxiety, fear and helplessness.

## Conclusion

While DAH work involved dealing with many difficult and undesired emotions prior to the Covid-19 pandemic, the findings in this article demonstrate that emotional challenges intensified during Covid-19. We argue that workplace emotion management is a collective interaction structured by feeling rules, and conducted through emotional reflexivity and the emotion work of multiple actors. WFH during Covid-19 made work-related emotion management and emotion work more challenging for DAHS. WFH reduced the physical spaces for DAHS to escape work-induced emotions, reduced spaces for engaging in emotional reflexivity and created additional strains such as protecting family members from the trauma of their work. In this context, DAHS and their managers performed emotion work – through leisure activities, comparing themselves to others, online conversations with colleagues and the gifting of ‘self-care’ kits – to enable DAHS to cope with their work-based emotions. This work-based emotion work acts as a plaster on workplace trauma in the absence of formal, sufficient, emotional support.

Emotion work is shown to be a key aspect of everyday work experience for DAHS, crucial for their ability to avoid the burnout often experienced by those in traumatic work (Babin et al., 2012; Crivatu et al., 2023; Helpingstine, 2021; Morran, 2008). Their emotion work is central to the social reproduction of the workforce – that is, the unpaid, often overlooked, work that sustains and reproduces the worker (Bhattacharya, 2017). This article adds to sociological understandings of work by separating the often conflated concepts of emotion work and emotional labour (Callahan and McCollum, 2002) to emphasise the presence and importance of emotion work of workers. Our findings demonstrate that emotion work conducted within DAHs during Covid-19 fills in for, and masks, the structural insufficiencies of DAH worker-wellbeing practices. We argue that examining emotion work can enable sociologists of work to identify the network of emotion management interactions that maintain worker wellbeing despite structural insufficiencies within existing worker-wellbeing practices. This can improve how we understand and better support worker wellbeing beyond DAHs.

Since the pandemic, WFH has become a ‘new normal’ in many workplaces (Raghavan et al., 2021). This article demonstrates the negative impact on DAHSs’ wellbeing, and required emotion work, that WFH during a pandemic can create. We recommend that fully home-based working is not suitable for those working in traumatic work. In the event of future lockdowns, or considerations to the long-term potential of remote working, we have a number of recommendations to ensure that DAHS are able to carry out their work in an emotionally sustainable way: (1) spaces should be provided for DAHS to work away from their home; (2) regular debriefs should be facilitated; (3) informal spaces for DAHS to continue to interact with colleagues should be created; and (4) there should be increased investment in staffing to reduce the workloads of DAHS.
